# Membrane simulation analysis using Voronoi tessellation

**DOI:** 10.1186/1758-2946-6-S1-O23

**Published:** 2014-03-11

**Authors:** Gunther Lukat, Björn Sommer, Jens Krüger

**Affiliations:** 1 Bio-/Medical Informatics Department, Bielefeld University, Bielefeld, NRW, 33615, Germany; 2 Theoretical Astrophysics Group, Hamburg Observatory, Hamburg, HH, 21029, Germany; 3 Applied Bioinformatics, University of Tübingen, Tübingen, BW, 72076, Germany

## 

The study of membranes and embedded proteins represents an advanced task in the field of molecular simulation. While nowadays a profound selection of sampling techniques, molecular topologies and theoretical approaches is available, the analysis of actual simulation data remains a difficult endeavour.

**Figure 1:**
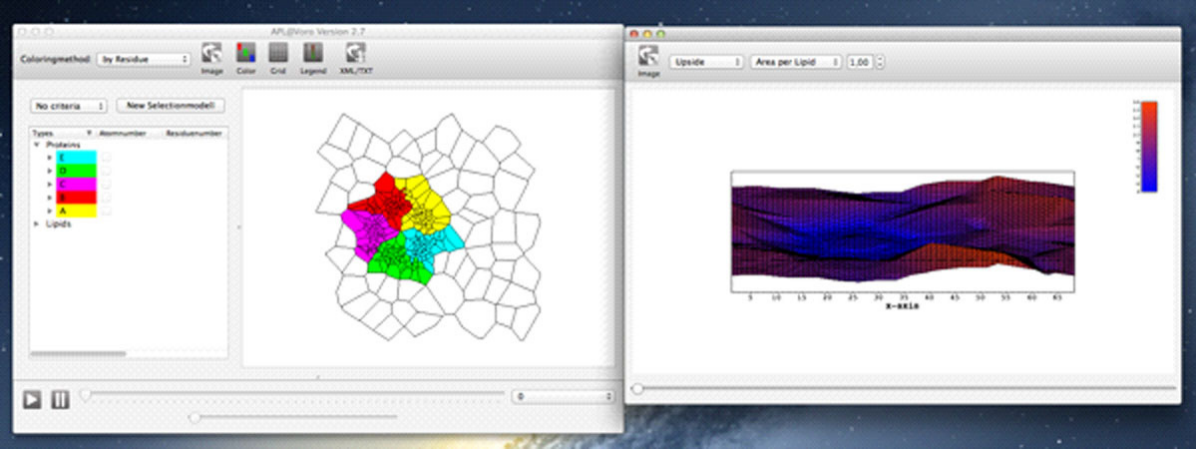
Pentamere of Vpu from HIV-1 in a POPC Bilayer. The Voronoi cells for the monomeres are colored, the cells for the lipids are left in white.

For homogeneous lipid bilayer simulations, the calculation of the bilayer thickness or area per lipid is directly accessible. For lipid mixtures, e.g. with cholesterol or embedded proteins, this is no longer the case. To face this challenge, APL@Voro has been developed [1]. The open-source and freely available graphical application is able to handle united-atom and coarse-grained trajectories generated with GROMACS [2]. Instructive, two-dimensional geometric representations of the lipid bilayer can easily be created based on Voronoi diagrams and Delaunay triangulations. The values, calculated on the geometric structures can be visualized in an interactive environment, plotted and exported to different file types (see Figure 1).

Even phase transitions within a bilayer can be tracked and visualised in an instructive and convenient way. APL@Voro represents a major improvement for the analysis of complex membrane simulations. The application is available at http://www.aplvoro.org.
